# Chronically critically ill patients: different behavior in severe sepsis and septic shock?

**DOI:** 10.1186/2197-425X-3-S1-A83

**Published:** 2015-10-01

**Authors:** DH Prevedello, A Rea-Neto, HA Teive, LA Tannous, RAO Deucher, MC Oliveira

**Affiliations:** Center for Study and Research in Intensive Care Medicine, Curitiba, Brazil; Federal University of Parana, Medical Clinic, Curitiba, Brazil; Federal University of Parana, Medical Research, Curitiba, Brazil; Hospital Cajuru, ICU, Curitiba, Brazil; Vita Hospital, ICU, Curitiba, Brazil

## Introduction

The chronically critically ill patient comes from an acute critical patient who survived the initial insult and did not recover their physiological baseline functions, depending on life support for weeks to months, especially on mechanical ventilation support. In literature, the most frequent definitions are the tracheostomy need or length of stay of at least 8 days in ICU.

## Objectives

Describe and compare clinical and epidemiological data of chronic and acute critically ill patients in severe sepsis and septic shock.

## Methods

Historical cohort. We analyzed 1293 patient charts from 4 intensive care units from January to December 2013. Patients were included if had at least 18 years old and had severe sepsis or septic shock with a minimum survival of 24 hours after the diagnosis. The cases were divided into 2 cohorts: the chronic critical patient (n = 100, 28.4%) and the acute critical patient (n = 252, 71.6%).

## Results

We analyzed 352 cases of severe sepsis (33.2%) and septic shock (66.8%). The average age was 65 (range 18 to 98), and 53.1% were men. The APACHE II average score was 20.9. Lung (58% vs 43.3% p = 0.017) and bloodstream (25% vs 7.5% p < 0.001) were the most prevalent sepsis focuses in the chronic cohort, but in the acute cohort were abdomen (22.2% vs 1% p < 0.001) and urine (15.5% vs 5% p = 0.007). There was no significant difference comparing SOFA, leukocytes, lactate and PCR, except the maximum respiratory frequency and the maximum temperature, these variables tended to have larger average values in chronically critical patients (RF 26ipm vs 24ipm p = 0.006 / T 36,8ºC vs 37,1ºC p = 0.001). When comparing life support need, there was an increased use of mechanical ventilation (86.9% vs 73.3% p = 0.01) and lower use of dobutamine (7% vs 17.5% p = 0.019) by chronically critically ill patients. The most common class of antibiotics in the chronic cohort was carbapenems (56.6% vs 33.3%, p < 0.001) and in the acute cohort it was anti-Pseudomonas penicillin (31% vs 16.2% p = 0.007). The largest duration of antibiotic therapy was on chronically critically ill patients (7.3 days vs 9.6 days p < 0.001). Analyzing the outcomes, the length of stay in the chronic group was higher (27.1 days vs 11.9 days p < 0.001). Also, the mortality rate until the 21st day of stay in ICU was higher in the acute cohort than in the chronic cohort, although the mortality rate in 60 days was higher in the chronic cohort (53% vs 40.1% p = 0,037).

## Conclusions

The profile of chronically critically ill is different from the acute patient. Chronic ill patients have more lung and blood infection as well as dependence on mechanical ventilation as life support. Besides, the chronically critically ill have a higher survival rate in the first 21 days in ICU; however, after that period, they begin to show a progressively higher mortality.
